# Nab-paclitaxel plus S-1 in advanced pancreatic adenocarcinoma (NPSPAC): a single arm, single center, phase II trial

**DOI:** 10.18632/oncotarget.21359

**Published:** 2017-09-28

**Authors:** Yan Shi, Sui Zhang, Quanli Han, Jie Li, Huan Yan, Yao Lv, Huaiyin Shi, Rong Liu, Guanghai Dai

**Affiliations:** ^1^ Medical Oncology Department 2, Chinese PLA General Hospital and Chinese PLA Medical School, Beijing, P.R. China; ^2^ Department of Medical Oncology, Dana-Farber Cancer Institute and Harvard Medical School, Boston, MA, USA; ^3^ Pathology Department, Chinese PLA General Hospital and Chinese PLA Medical School, Beijing, P.R. China; ^4^ Department of Hepatobiliary and Pancreatic Surgical Oncology, Chinese PLA General Hospital and Chinese PLA Medical School, Beijing, P.R. China

**Keywords:** nab-paclitaxel, S-1, objective response rate, survival, advanced pancreatic adenocarcinoma

## Abstract

This single-arm, phase II trial is to investigate efficacy and safety of nab-paclitaxel plus S-1 as first-line treatment in advanced pancreatic cancer. Nab-paclitaxel was administered at 120 mg/m^2^ intravenously on day 1 and 8, S-1 was given twice a day orally on day 1-14 of each 21-day cycle, for 6 cycles. The primary endpoint was objective response rate (ORR), the secondary endpoints were progression-free survival (PFS), overall survival (OS) and safety. The ORR in intent-to-treat population (N=60) by either blinded independent review (BIR) or investigator assessment was 50.0%. Median PFS (mPFS) by BIR and median OS (mOS) were 5.6 months (95%CI, 4.6 to 6.6 m) and 9.4 months (95%CI, 8.0 to 10.8m), respectively. The most common grade 3 or 4 toxicities were leukopenia/neutropenia (35%) and fatigue (8.3%). Subgroup analyses based on BIR showed a remarkable ORR (>70%) was achieved in patients with female gender, ≥ 50% decline from baseline CA19-9, and developed grade 3 or 4 leukopenia/neutropenia. Remarkable survival benefit was statistically significant in female (mPFS: 7.7m, mOS: 18.2m), ≥ 50% decline from baseline CA19-9 (mPFS: 6.8m, mOS: 11.8m), objective responders (mPFS: 6.9m, mOS: 12.2m), and ECOG of 0 at baseline (mPFS: 7.5m, mOS: 16.1m). Nab-paclitaxel plus S-1 showed encouraging ORR and manageable toxicities, which is an effective alternative treatment regimen for advanced pancreatic cancer. (https://clinicaltrials.gov/ number, NCT02124317)

## INTRODUCTION

Pancreatic adenocarcinoma (PAC) is the seventh leading cause of cancer-related death worldwide [1]. In 2015, the estimated new cases and associated deaths of PAC were 90,100 and 79,400, respectively, in China [2]. The majority of PAC patients have unresectable advanced disease (locally advanced or metastatic) and lack of effective therapeutic options at the time of diagnosis [3], which leave patients miserable life expectancy with a 5-year survival rate less than 5% [4, 5]. Gemcitabine monotherapy was the only approved first-line treatment in patients with advanced PAC for about 15 years before 2011, however the objective response rate (ORR) was 4-9%, whereas the median overall survival (OS) was 5.4-7 months, and 1-year survival rate was 17-23%[6–9].

Recent two large, randomized phase III studies showed promising effects in metastatic PAC treated by FOLFIRINOX (5-FU, leucovorin, irinotecan, and oxaliplatin) or nab-paclitaxel plus gemcitabine [8, 10]. However, the confirmed ORRs of these two regimens were 23-34%, which still unmet clinical expectation, in addition there were significantly increased hematologic adverse events of grade 3 or higher. S-1 is an oral fluoropyrimidine derivative, which demonstrated effectiveness and good tolerability in gastric and some other cancers. In GEST and JASPAC01 studies, S-1 monotherapy demonstrated comparable or even superior clinical benefit in treatment of advanced and postoperative PAC compared to gemcitabine [11, 12]. Meanwhile S-1 had less adverse events, especially in neutropenia compared to gemcitabine (≥ grade 3: 8.8% vs. 41% in GEST; 13% vs. 72% in JASPAC01). Therefore, S-1 is theoretically a favorable alternative partner for nab-paclitaxel given its non-inferior antitumor activity and better tolerability compared to gemcitabine in treatment of PAC. This phase II study was designed to investigate the efficacy and safety of nab-paclitaxel in combination with S-1 as the first-line treatment in patients with locally advanced and metastatic PAC.

## RESULTS

### Patient characteristics

Between April 2014 and October 2016, a total of 60 patients were enrolled. Fifty-five patients (91.7%) had metastatic disease and 5 patients had locally advanced PAC. Thirty-one patients (51.7%) had an Eastern Cooperative Oncology Group (ECOG) performance status of 0, and all patients received at least 2 cycles of nab-paclitaxel in combination with S-1. Three patients had history of prior surgical resection including one treated with gemcitabine alone as adjuvant therapy more than six months before enrollment, and the other two did not receive adjuvant therapy due to hypoproteinemia and patient's personal decision. Baseline characteristics of patients are shown in Table 1.

**Table 1:** Patient demographics and disease characteristics at baseline

Characteristic	No.	%
Age, years		
Median (Range)	56 (34 - 74)	
Sex		
Male	42	70.0
Female	18	30.0
ECOG performance status		
0	31	51.7
1	29	48.3
Diabetes		
Yes	12	20.0
No	48	80.0
Tumor grade differentiation		
Well/Moderate	43	71.7
Moderate-poor/Poor	17	28.3
Stage		
Locally advanced	5	8.3
Metastatic	55	91.7
Location of primary tumor		
Head/neck of pancreas	17	28.3
Body/tail of pancreas	43	71.7
Prior surgical resection ^*^	3	5.0
Site of metastatic disease		
Abdomen/peritoneal	9	15.0
Liver	47	78.3
Lung	8	13.3
Others	3	5.0
Liver only	24	40.0
No. of metastatic disease		
0	5	8.3
1	30	50.0
2	15	25.0
≥ 3	10	16.7
CA19-9 baseline levels, No.		
Normal	8	13.3
Elevated	52	86.7
CA19-9 baseline, U/mL ^#^		
Median (Range)	2189 (1 - >20,000)	

Abbreviation: ECOG, Eastern Cooperative Oncology Group.

^*^ Three patients had history of prior surgical resection including one treated with gemcitabine alone as adjuvant therapy more than six months before enrollment.

^#^ Normal CA19-9 levels are ≤ 35 U/mL in our hospital.

### Efficacy

**Figure 1:**
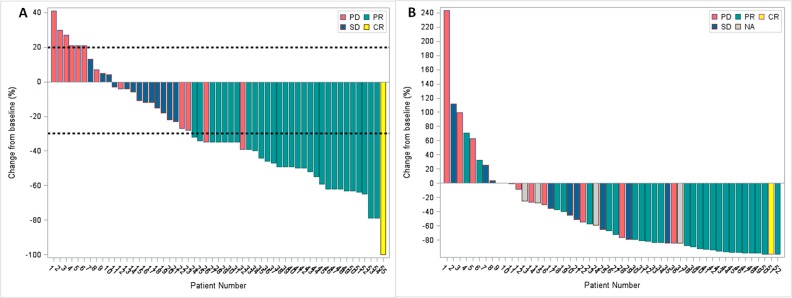
Waterfall plots, the color keys indicate the best overall response by blinded independent review **(A)** the best percentage change in target lesion determined by RECIST 1.1 for all evaluable patients (N=55), and the dashed lines at 20% and -30% represent the progressive disease and partial response, respectively; **(B)** the best percentage change of CA19-9 in evaluable patients who had an elevated CA19-9 at baseline (N=52). Abbreviation: CR, completed response; PR, partial response; SD, stable disease; PD, progressive disease; NA, not available.

Five patients had no response evaluation including 4 lost follow-up and 1 worsening ECOG performance status after 2 cycles of treatment. The ORR in intention-to-treat (ITT) population (N=60) based on investigator's assessment was 50% (2 complete response [CR], 3.3%; and 28 partial response [PR] 46.7%) (Table 2), and the disease control rate (DCR) was 81.7%. The ORR and DCR by blinded independent review (BIR) were 50% (1 CR, 1.7%; and 29 PR, 48.3%) and 71.7%, respectively. A water fall plot of the best response based on independent imaging assessment of nab-paclitaxel plus S-1 treatment is shown in Figure 1A, whereas the best response was defined as the best target lesion(s) response recorded from the start to the end of the treatment. In the 55 evaluable patient population, the ORR and DCR were 54.5%, 89.1% and 54.5%, 78.2% by investigator and BIR, respectively.

**Table 2:** Response rates per RECIST 1.1 criteria in patients treated with nab-paclitaxel plus S-1 (ITT population)

Tumor response, No. (%)	Best response by investigator review	Best response by independent review
Complete response^#^	2 (3.3)	1 (1.7)
Partial response^#^	28 (46.7)	29 (48.3)
Stable disease	19 (31.7)	13 (21.7)
Progressive disease	6 (10.0)	12 (20.0)
Not assessable^*^	5 (8.3)	5 (8.3)
Objective response rate	30 (50.0)	30 (50.0)
Disease control rate	49 (81.7)	43 (71.7)

Abbreviation: ITT, Intention-to-Treat.

^#^ Complete response and partial response were all confirmed at least 4 weeks apart according to RECIST 1.1 criteria.

^*^ Five patients had no response evaluation due to lost follow-up after 2 cycles of treatment in 4 patients and one had worsening Eastern Cooperative Oncology Group performance status.

In 52 patients with elevated carbohydrate antigen 19-9 (CA19-9) at baseline, 32 (59.6%) had ≥ 50% decline from baseline CA19-9. The association between maximum percentage change in CA19-9 levels of the patients and the confirmed best overall response according to the BIR is displayed on a waterfall plot (Figure 1B).

### Treatment exposure and safety

The median treatment cycle was 4 (range, 2 to 6 cycles, total 275 cycles). Eleven patients (18.3%) required dose reduction of one or both drugs due to adverse events, among which 7 patients had reduction in the nab-paclitaxel dose and 8 patients had reduction in the S-1 dose. The median relative dose intensities of nab-paclitaxel and S-1 were both 100%. Twenty-seven patients (45%) completed 6 cycles of planned treatment, while 33 patients discontinued treatment due to disease progression (N=15), patients’ refusal (N=11), lost follow-up (N=4) and adverse event (N=3). Total 23 patients received S-1 maintenance treatment, whereas 16 patients were after 6 cycles, while the other 5 and 2 patients had treatment response but started maintenance treatment after 4 and 5 cycles due to patients’ desire or adverse event.

The frequencies of common hematological and non-hematological adverse events (noted by ≥ 10% of patients) are listed in Table 3. The most commonly reported adverse events including all grades were leukopenia/neutropenia (88.3%), sensory neuropathy (78.3%), nausea/vomiting (71.7%) and anemia (70.0%). In total, 18 (30%) and 7 (11.7%) patients experienced ‘at least once’ grade 3 or grade 4 (leukopenia or neutropenia only) adverse events respectively, whereas 17 grade 3 and 5 grade 4 were successfully resolved after supportive management. The most common grade 3 or 4 treatment related toxicities were leukopenia/neutropenia (35%), fatigue (8.3%), anemia (6.7%) and sensory neuropathy (5%). Of the 3 patients discontinued treatment due to unresolvable adverse events, 2 experienced grade 4 neutropenia and grade 3 mucositis, and the treatment had to be terminated after 4 cycles due to decreasing ECOG performance score after treatment delay and dose reduction. Another had grade 3 neutropenia and fatigue, similarly the treatment had to be terminated due to worsening fatigue after dose reduction before cycle 3. One patient died within 30 days after the last treatment due to disease progression rather than treatment-related toxicities (Table 3).

**Table 3:** Treatment-related adverse events occurring in ≥ 10% of patients in ITT population (N=60)

Adverse event	Grade 1		Grade 2		Grade 3		Grade 4		Total	
	No.	%	No.	%	No.	%	No.	%	No.	%
Hematologic										
Anemia	19	31.7	19	31.7	4	6.7	0	0	42	70.0
Leukopenia/Neutropenia	14	23.3	18	30.0	14	23.3	7	11.7	53	88.3
Thrombocytopenia	13	21.7	3	5.0	0	0	0	0	16	26.7
Nonhematologic										
Fatigue	8	13.3	4	6.7	5	8.3	0	0	17	28.3
Diarrhea	3	5.0	2	3.3	1	1.7	0	0	6	10.0
AST/ALT elevated	12	20.0	3	5.0	0	0	0	0	15	25.0
Nausea/ Vomiting	35	58.3	6	10.0	2	3.3	0	0	43	71.7
Hand-foot syndrome	9	15.0	4	6.7	0	0	0	0	13	21.7
Mucositis	6	10.0	1	1.7	2	3.3	0	0	9	15.0
Sensory neuropathy	22	36.7	22	36.7	3	5.0	0	0	47	78.3

Abbreviation: ITT, Intention-to-Treat; AST, Aspartate transaminase; ALT, Alanine transaminase.

### Second-line therapy

Second-line therapy was administered in 18 patients (30%) in our cohort. The regimens were gemcitabine alone in 7 patients and gemcitabine-based combination in 11 patients.

### Survival analysis

**Figure 2:**
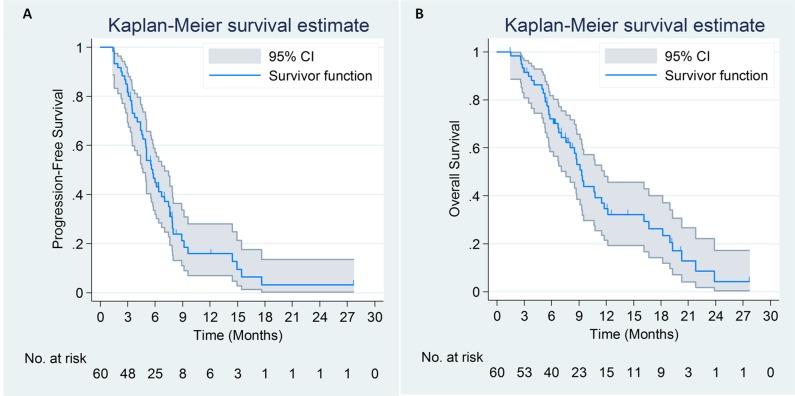
Progression-free survival **(A)** by blinded independent review and overall survival curves **(B)** of 60 patients with advanced pancreatic cancer treated with nab-paclitaxel plus S-1.

By March 1, 2017, 45 patients died and 4 patients lost follow-up. Median progression-free survival (mPFS) according to the investigator assessment and BIR were 5.8 months (95% CI, 4.9 to 6.7m) and 5.6 months (95% CI, 4.6 to 6.6m), respectively. Median OS (mOS) time for the study cohort was 9.4 months (95% CI, 8.0 to 10.8m) (Figure 2).

### Subgroup analysis

We further stratified analyses on the subgroups to distinguish the population potentially received more benefit from nab-paclitaxel in combination with S-1. Female (76%), patients with ≥ 50% decline from baseline CA19-9 (77%), and those had grade 3 or 4 leukopenia or neutropenia (75%) gained better ORR based on BIR (Table 4).

**Table 4:** Subgroup analyses of objective response rates by blinded independent review and investigator review

Characteristic	No. (%)	ORR by independent review (95% CI)	P	ORR by investigator review (95% CI)	P
Age			0.35		0.49
≤ 56	30 (50.0)	48% (31% - 66%)		59% (41% - 75%)	
> 56	30 (50.0)	61% (42% - 76%)		50% (33% - 67%)	
Sex			0.03		0.03
Male	42 (70.0)	45% (30% - 60%)		45% (30% - 60%)	
Female	18 (30.0)	76% (53% - 90%)		76% (53% - 90%)	
ECOG performance status			0.49		0.69
0	31 (51.7)	50% (33% - 67%)		57% (39% - 73%)	
1	29 (48.3)	59% (41% - 75%)		52% (34% - 69%)	
Diabetes			0.50		0.50
Yes	12 (20.0)	64% (35% - 85%)		45% (21% - 72%)	
No	48 (80.0)	52% (38% - 66%)		57% (42% - 70%)	
Tumor grade differentiation			0.27		0.62
Well/Moderate	43 (71.7)	50% (35% - 65%)		53% (37% - 67%)	
Moderate-poor/Poor	17 (28.3)	67% (42% - 85%)		60% (36% - 80%)	
Stage			0.49		0.49
Locally advanced	5 (8.3)	40% (12% - 77%)		40% (12% - 77%)	
Metastatic	55 (91.7)	56% (42% - 69%)		56% (42% - 69%)	
Location of primary tumor			0.05		0.18
Head of pancreas	17 (28.3)	33% (15% - 58%)		40% (20% - 64%)	
Body or tail of pancreas	43 (71.7)	63% (47% - 76%)		60% (45% - 74%)	
CA19-9 baseline levels			0.78		0.29
Normal	8 (13.3)	50% (22% - 78%)		38% (14% - 69%)	
Elevated	52 (86.7)	55% (41% - 69%)		57% (43% - 70%)	
≥ 50% decline from baseline CA19-9^*^			<0.001		0.003
Yes	32 (61.5)	77% (59% - 88%)		73% (56% - 86%)	
No	20 (38.5)	18% (6% - 41%)		29% (13% - 53%)	
Leukopenia/Neutropenia^#^			0.02		0.02
Grade 0-2	39 (65.0)	43% (28% - 59%)		43% (28% - 59%)	
≥ Grade 3	21 (35.0)	75% (53% - 89%)		75% (53% - 89%)	

Abbreviation: ECOG, Eastern Cooperative Oncology Group; ORR, objective response rate.

^*^ The change of CA19-9 after treatment was evaluated in 52 patients with elevated CA19-9 baseline levels.

^#^ Grade of leukopenia/Neutropenia was assessed according to the National Cancer Institute Common Terminology Criteria for Adverse Events (version 4.0).

In terms of subgroup survival analyses, median PFS by BIR and median OS in female were 7.7 and 18.2 months compared to 5.0 months (P=0.002) and 8.5 months (P=0.002) in male, respectively. The patients with baseline ECOG performance score of 0 had longer mPFS and mOS than those of 1 (mPFS 7.5m vs 5m, P=0.005; mOS 16.1m vs 7.6m, P=0.01). As expected, the favorable responders (PR or CR) was associated with better PFS and OS compared to non-responders (SD+PD) assessed by BIR (mPFS 6.9m vs 3.3m, P=0.002; mOS: 12.2m vs 5.7m, P<0.001). Similarly, the patients with ≥ 50% decline from baseline CA19-9 also had longer PFS (6.8m vs 3.7m, P=0.02) and OS (11.8m vs 5.7m, P=0.02) compared to the rest.

## DISCUSSION

The treatment response of nab-paclitaxel and S-1 in this phase II trial met and exceeded our expectation in patients with advanced PAC. The remarkable ORR of 50% in ITT population, supported by BIR, was far exceeded our hypothesis of achieving 20%, and better than the historic data (ORR less than 35%) in the previous studies with cytotoxic regimens in advanced or metastatic PAC [8–11]. With respect to the secondary endpoints (safety, PFS and OS), this two-agent combination also showed good tolerability with a median PFS of 5.6 months by BIR and a median OS of 9.4 months, which was comparable to other combinations of two-cytotoxic-drug regimens, such as nab-paclitaxel with gemcitabine and gemcitabine with S-1 [10–12].

Although this was a single arm trial, the outstanding ORR suggested it is worth for investigators to put more attention on this treatment combination, and further investigation should be conducted in PAC. Recently, some studies revealed synergistic activity of the combination of nab-paclitaxel and S-1 in PAC from bench to bedside, which provided sufficient scientific base of our study. Suenaga M. et al. reported that S-1 and nab-paclitaxel had a synergistic effect *in vitro* and showed greater efficacy than monotherapy *in vivo* [13]. The possible mechanism of this combination may be due to the improvement of the stromal composition (stromal depletion) and tumor angiogenesis in the subcutaneous model. Li JA. et al. further proved the efficacy of this combination in patient-derived pancreatic cancer xenograft (PDX) mouse models [14]. They found that S-1 and nab-paclitaxel showed significantly better antitumor activity than monotherapy, and this combination played a role in stroma depletion and increasing vascularization in PDX models. In fact, the efficacy and safety of the same treatment combination was established in the advanced breast cancer and gastric cancer [15, 16]. Furthermore, the combination of nab-paclitaxel with simplified leucovorin and fluorouracil showed good tolerability and efficacy (over 50% patients were progression-free at 4 months) as first-line chemotherapy for patients with metastatic pancreatic cancer in a recent phase II trial [17]. Given these preclinical and preliminary clinical data, the combination of nab-paclitaxel and S-1 could theoretically be an option for pancreatic cancer.

Our subgroup analyses showed the remarkable ORR (>70%) was achieved in patients with female gender, ≥ 50% decline from baseline CA19-9, and developed grade 3 or 4 leukopenia or neutropenia. Remarkable survival benefit was statistically significant in female (mPFS: 7.7m, mOS: 18.2m), ≥ 50% decline from baseline CA19-9 (mPFS: 6.8m, mOS: 11.8m), Response Evaluation Criteria in Solid Tumor (RECIST) responders (mPFS: 6.9m, mOS: 12.2m), and ECOG of 0 at baseline (mPFS: 7.5m, mOS: 16.1m). Early in 1998, Micheli A. et al. reported female patients typically survive longer in pancreatic cancer [18]. Subsequently in a recent retrospective study, Hohla F. et al. found female gender was a protective factor in FOLFIRINOX treatment of unresectable pancreatic cancer [19]. Gender difference in cancer susceptibility and prognosis may be associated with different role of hormones as the promoting action of androgens and protective action of estrogens on pancreatic carcinogenesis [20, 21]. Besides the ECOG status, gender balance should be taken into consideration for randomize balance in future phase III trials. In patients developed grade 3 or 4 leukopenia or neutropenia, we highly suggest the patient should be encouraged for continuous treatment in case of reversal of the adverse event.

There were limitations in our study. This was a single-center, single-arm, nonrandomized trial. Another limitation of our study was that we did not test the levels of thymidylate synthase (TS), orotate phosphoribosyl-transferase (OPRT) and dihydropyrimidine dehydrogenase (DPD), which were considered potentially being associated with the efficacy and safety of S-1 [22]. Due to potential difference between Westerners and Asians in pharmacokinetics and pharmacodynamics of S-1, it is recommended that the plasma concentration, and effectiveness and toxicity related biomarkers (TS and DPD) should be monitored and examined if the similar regimen is applied to Western patients [23]. Emerging evidence showed that metabolic response was associated with greater efficacy and longer survival [24], however we did not perform positron emission tomography (PET) for metabolic response assessment in our protocol. Limited sample size and mixed 5 cases of locally advanced PAC into metastatic PAC were also our limitations. However, this was the first study to discover the promising favorable response of the combination of nab-paclitaxel with S-1 in advanced PAC.

In conclusion, nab-paclitaxel plus S-1 demonstrated a remarkable antitumor activity with good tolerability and manageable toxicity as the first-line treatment in patients with advanced PAC in Chinese population. Given the convenient administration of S-1, we found this regimen was more manageable practically. The significant ORR made us believe this treatment regimen is potentially an effective alternative for those with gemcitabine allergy and poor compliance with nab-paclitaxel and gemcitabine or FOLFIRINOX treatment. Given the promising efficacy and safety of nab-paclitaxel plus S-1, a larger randomized phase III trial is warranted.

## MATERIALS AND METHODS

### Patients

The study protocol was approved by the ethics committee of the Chinese PLA General Hospital (No. S2014-031-01), and all procedures were in accordance with the ethical standards of the responsible committee on human experimentation (institutional and national) and with the Declaration of Helsinki and Good Clinical Practice guidelines. All patients provided written informed consent before entering the study.

Patients eligible for enrollment were: a) adults no less than 18 years of age; b) histologically or cytologically confirmed locally advanced or metastatic PAC; c) ECOG performance status of 0 or 1 with life expectancy no less than 12 weeks; d) having at least one measurable disease by computed tomography (CT) as defined in the RECIST version 1.1 [25]; e) no previous 5-fluorouracil or gemcitabine treatment unless they were used in the adjuvant setting for radiation therapy no less than 6 months and no lingering toxicities were present before enrollment; f) adequate bone marrow (absolute neutrophil count ≥ 1.5 μL and platelets ≥ 100,000 μL) and liver functions (bilirubin ≤ 1.5 times the upper limit of the normal range), and normal renal function.

Patients were excluded from the enrollment if they: a) were endocrine or acinar pancreatic carcinoma; b) had history of other malignancy; c) had uncontrolled brain metastasis or mental illness; d) had uncontrolled concomitant medical illnesses (e.g., active infection, cardiac disease and sever peripheral neuropathy), e) were pregnancy or breast-feeding.

### Study design and treatment

This study was a prospective, single center, single arm, open label phase II clinical trial conducted in PLA General Hospital in China. Nab-paclitaxel was administered at 120 mg/m^2^ in 30 to 40 minute intravenously on day 1 and 8, S-1 was given twice a day orally at a dose according to the body surface area (BSA) (< 1.25 m^2^, 80 mg/d; ≥ 1.25 to < 1.5 m^2^, 100 mg/d; ≥ 1.5 m^2^, 120 mg/d) on days 1 through 14 of each 21-day cycle. Given the dosage of nab-paclitaxel was reduced in 41% patients and the median relative dose intensity of nab-PTX was 81% in MPACT study, the lower dose of nab-paclitaxel was used with dose intensity at 80 mg/m^2^ per week in this study. Six cycles of chemotherapy were planned for patients who had a response. The treatment discontinued if patient had disease progression, unacceptable toxicity, or request of stop treatment. In patients with clinical benefit after completion of 6-cycle treatment or treatment discontinued, S-1 monotherapy was allowed to be given as maintenance therapy at investigator's discretion according to patients’ desire and ECOG performance status. Patients were followed up every 2 months until death.

Dose interruption or discontinuation was permitted in case of grade 3 to 4 hematologic or grade 3 nonhematologic toxicity, and supportive management was instituted. Dose adjustments of either nab-paclitaxel or S-1 were allowed but no more than twice for each patient, whereas nab-paclitaxel could be adjusted to 100 mg/m^2^ and 80 mg/m^2^subsequently, and S-1 could reduce by 20mg per day every time if reversal of toxicity to grade 1 or better within 28 days; otherwise, the treatment was terminated.

### Assessments

Clinical and laboratory evaluations, including the change of serum CA19-9, were performed at baseline and every 3 weeks. Adverse events were assessed by the investigators before each cycle according to the National Cancer Institute Common Terminology Criteria for Adverse Events (version 4.0). Tumor responses including CR, PR, stable disease (SD), or progressive disease (PD) were evaluated on CT or magnetic resonance imaging (MRI) according to RECIST 1.1 at baseline and every 6 weeks (± 7 days) by the investigators and blinded independent reviewer (X.M.). 18F-fluorodeoxygluscose PET scan was used to confirm CR.

### Statistical analysis

The primary endpoint was ORR, the secondary endpoints were PFS, OS and safety. The ORR was calculated as the number of patients with CR or PR divided by the total number of patients, whereas CR or PR was confirmed on imaging assessment 4 weeks apart per RECIST 1.1 criteria. OS and PFS were calculated from the date of the treatment initiation until the date of death or the date of documentation of disease progression or death in patients without disease progression, whichever occurred first. The censoring date was the last date of available follow-up.

The sample size was determined using a one stage design, with the assumption that an increase of ORR from 8% (the ORR of gemcitabine alone based on prior studies of first-line treatment in metastatic PAC) to 20% with a significance level of 0.05 and a power of 80%. Considering a 10% drop off rate, total 60 patients were required.

Demographic and baseline characteristics, response rates and safety observations were summarized using descriptive statistics. The χ2 test was performed to compare the ORR between different subgroups. PFS and OS were estimated using Kaplan-Meier method, and the difference in survival between subgroups was assessed by the log-rank test. The statistical significance was defined as a two-sided P<0.05. All statistical analyses were conducted by a statistician (S.Z.) using SAS software (version 9.4; SAS Institute, Cary, North Carolina).

## Abbreviations

BIR: blinded independent review
CA19-9: carbohydrate antigen 19-9
CR: complete response
DCR: disease control rate
DPD: dihydropyrimidine dehydrogenase
ECOG: Eastern Cooperative Oncology Group
ITT: intention-to-treat
OPRT: orotate phosphoribosyl-transferase
ORR: objective response rates
OS: overall survival
PAC: pancreatic adenocarcinoma
PDX: patient-derived xenograft
PET: positron emission tomography
PD: progressive disease
PFS: progression-free survival
PR: partial response
SD: stable disease
TS: thymidylate synthase
